# Redoxing PTPN22 activity

**DOI:** 10.7554/eLife.79125

**Published:** 2022-05-19

**Authors:** Magdalena Shumanska, Ivan Bogeski

**Affiliations:** 1 Molecular Physiology Division, Institute of Cardiovascular Physiology, University Medical Center, Georg-August University Göttingen Germany

**Keywords:** inflammation, T cells, PTPN22, Redox, Mouse

## Abstract

The oxidative state of a critical cysteine residue determines the enzymatic activity of a phosphatase involved in T-cell immune responses.

**Related research article** James J, Chen Y, Hernandez CM, Forster F, Dagnell M, Cheng Q, Saei AA, Gharibi H, Lahore GF, Åstrand A, Malhotra R, Malissen B, Zubarev RA, Arnér ESJ, Holmdahl R. 2022. Redox regulation of PTPN22 affects the severity of T-cell-dependent autoimmune inflammation. *eLife* **11**:e74549. doi: 10.7554/eLife.74549.

A precisely tuned immune system is tremendously important for rapidly sensing and eliminating disease-causing pathogens and generating immunological memory. At the same time, immune cells need to be able to recognize the body’s own cells and distinguish them from foreign invaders. Even small dysregulations can result in the immune system attacking organs and tissues in the body by mistake, leading to conditions known as autoimmune diseases.

The incidence of autoimmune diseases worldwide has increased in recent years, leading scientists to investigate how genetic and environmental factors contribute to these pathologies (Ye et al., 2018). Amongst other findings, research has shown that an enzyme called PTPN22 (short for protein tyrosine phosphatase non-receptor type 22) is a risk factor in multiple autoimmune disorders, including rheumatoid arthritis, diabetes and systemic lupus erythematosus. PTPN22 prevents the overactivation of T-cells (cells of the adaptive immune system) by removing phosphate groups from phosphorylated proteins that are part of the T-cell receptor (TCR) signaling pathway (Figure 1; Bottini et al., 2006; Fousteri et al., 2013; Tizaoui et al., 2021).

**Figure 1.**
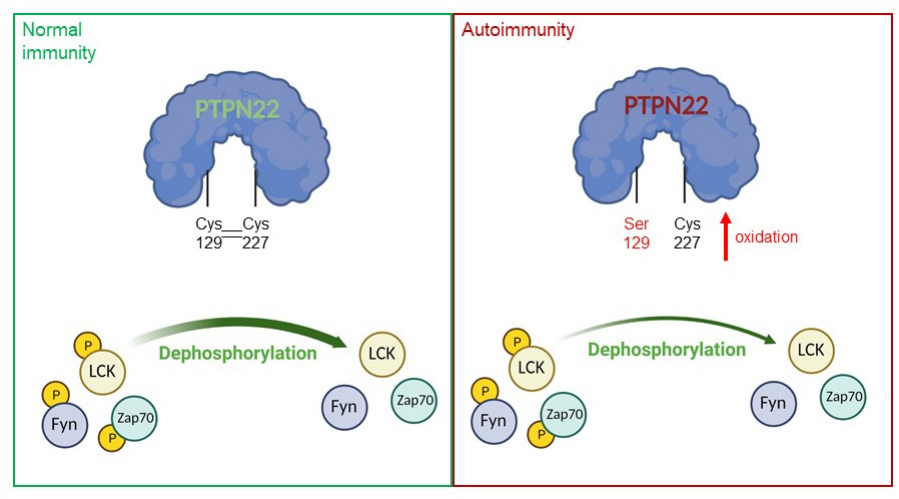
A model of PTPN22 redox regulation and its effect on T-cell activity. In normal immunity, wildtype PTPN22 (left, blue protein with green lettering) is able to efficiently remove phosphate groups (yellow circles) from proteins downstream of the T-cell receptor (TCR), including LCK, Fyn and Zap70. Dephosphorylation inactivates these proteins, reducing T-cell activity. In this state, two PTPN22 cysteine residues (at positions 129 and 227) form a disulfide bond, which influences the redox state and the activity of the enzyme. If PTPN22 is mutated so that cysteine 129 becomes a serine (right, blue protein with red lettering, with the mutant serine residue shown in red), the disulfide bond cannot form, and the phosphatase becomes more sensitive to deactivation by oxidation. The mutant version of the phosphatase is also less efficient at dephosphorylating proteins, which increases TCR signaling and inflammation, leading to autoimmunity.

Figure created using BioRender

Activation of the T-cell receptor is followed by the production of reactive oxygen species (ROS), highly reactive by-products of molecular oxygen, which can oxidize other molecules, including proteins. It is now clear that ROS have important roles in T-cell activation and that defects in ROS production may alter the immune system's responses (Simeoni and Bogeski, 2015; Kong and Chandel, 2018). However, high levels of ROS can also cause oxidative stress, leading to impaired cell activity and even death. Therefore, T-cells must optimally balance ROS production through antioxidative mechanisms and enzymes such as thioredoxin (Patwardhan et al., 2020).

Redox reactions (oxidation and its reverse reaction known as reduction) regulate many proteins, including phosphatases (Tonks, 2005), although how oxidation and reduction modulate PTPN22 activity remained unclear. Now, in eLife, Rikard Holmdahl and colleagues based in Sweden, China, Australia, Austria, France, Russia, Hungary and the United States – including Jaime James (Karolinska Institute) as first author – report that a non-catalytic cysteine may play an important role in the redox regulation of PTPN22 (James et al., 2022). Notably, this regulation was found to modulate inflammation in mouse models with severe autoimmunity.

The team genetically engineered mice that carried a mutated version of PTPN22, in which a non-catalytic cysteine at position 129 was replaced with a serine, preventing that residue from forming a disulfide bond with the catalytic cysteine at position 227 responsible for the enzymatic activity of PTPN22. Notably, this approach was based on a study in which the crystal structure of PTPN22 was examined and an atypical bond was observed between the non-catalytic cysteine at position 129 and the catalytic cysteine residue (C227; Orrú et al., 2009). In vitro experiments performed by James et al. revealed that the mutant enzyme was more sensitive to inhibition by oxidation than its wildtype counterpart. Interestingly, the results also showed that the mutant PTPN22 was less efficient at performing its catalytic role, and that it was less responsive to re-activation by antioxidant enzymes, such as thioredoxin.

To further test the role of cysteine 129 in PTPN22 redox regulation, James et al. used a mouse model that expressed the mutant protein and was susceptible to rheumatoid arthritis. These mice exhibited higher levels of inflammation in response to T-cell activation, which would be expected in animals that cannot downregulate TCR signaling. The mice also displayed more severe symptoms of arthritis, consistent with high immune activity. These effects were not observed when the experiment was repeated in mice that fail to produce high levels of ROS in response to TCR activation, confirming that the initial observations depend on the redox state of PTPN22.

Finally, James et al. performed in vitro experiments on T-cells isolated from mice carrying the mutant PTPN22. They found that when these cells became activated, the downstream targets of PTPN22 showed an increased phosphorylation status, consistent with lower PTPN22 activity.

Taken together, the elegant study of James et al. shows that cysteine 129 is critical for the redox regulation of PTPN22, and that its mutation impacts T-cell activity and exacerbates autoimmunity in mice (Figure 1). What still remains to be determined is why the mutant enzyme has lower catalytic activity, which may be due to the mutation affecting the structural conformation of PTPN22. Additionally, it will be important to assess other cysteines in PTPN22 to determine whether they are also partly involved in its redox regulation.

Understanding how the redox state of PTPN22 regulates the activity of T-cells may help researchers to develop new therapies for treating autoimmune diseases.
